# Supplementary material for: Urine Cell-Free MicroRNAs in Localized Prostate Cancer Patients
Source: Cancers (Basel). 2022 May 12;14(10):2388. doi: 10.3390/cancers14102388 (PMC9139357; doi:10.3390/cancers14102388)
Supplement: Supplementary file 1 [file cancers-14-02388-s001.zip › cancers-1690887-supplementary.pdf]

cancers

MDPI

*Supplementary Materials*

# Urine Cell-Free MicroRNAs in Localized Prostate Cancer Patients

**Yoko Koh [1,2], Matias A. Bustos [1], Jamie Moon [1], Rebecca Gross [1,2], Romela Irene Ramos [1], Suyeon Ryu [3], Jane Choe [2], Selena Y. Lin [4], Warren M. Allen [5], David L. Krasne [5], Timothy G. Wilson [2] and Dave S. B. Hoon [1,3,*]**

[1] Department of Translational Molecular Medicine, Saint John's Cancer Institute (SJCI), Providence Saint John's Health Center (SJHC), Santa Monica, CA 90404, USA; yoko.koh@providence.org (Y.K.); Matias.Bustos@providence.org (M.A.B.); jamie.moon@providence.org (J.M.); Rebecca.Gentry@providence.org (R.G.); Romela.Ramos@providence.org (R.I.R.)
[2] Department of Urology and Urologic Oncology, SJCI, Providence SJHC, Santa Monica, CA 90404, USA; jane.choe@providence.org (J.C.); Timothy.Wilson@providence.org (T.G.W.)
[3] Genome Sequencing Center, SJCI, Providence SJHC, Santa Monica, CA 90404, USA; Suyeon.Ryu@providence.org
[4] JBS Science Inc., Doylestown, PA 18902, USA; selenayl@gmail.com
[5] Division of Surgical Pathology, Providence SJHC, Santa Monica, CA 90404, USA; Warren.Allen@providence.org (W.M.A.); David.Krasne@providence.org (D.L.K.)
* Correspondence: Dave.Hoon@providence.org

**Simple Summary:** Urine cell-free microRNAs (cfmiRs) are promising biomarkers for the detection of prostate cancer (PCa) and may replace or complement prostate-specific antigen screening. This pilot study aims to demonstrate the diagnostic utility of urine cfmiRs for early-stage PCa using a robust microRNA (miR) panel based on next-generation sequencing. We assessed urine, plasma, and formalin-fixed paraffin-embedded tumor tissue samples obtained from patients diagnosed with pT2 PCa. Differentially expressed miRs were found in urine, plasma, and tumor samples obtained from PCa patients. Through bioinformatic analysis, several miRs were found as potential cfmiRs with utility for the detection of PCa. Our results showed that specific cfmiRs in urine samples from PCa patients may have potential utility in the detection of early-stage PCa.

**Abstract:** Prostate cancer (PCa) is the most common cancer in men. Prostate-specific antigen screening is recommended for the detection of PCa. However, its specificity is limited. Thus, there is a need to find more reliable biomarkers that allow non-invasive screening for early-stage PCa. This study aims to explore urine microRNAs (miRs) as diagnostic biomarkers for PCa. We assessed cell-free miR (cfmiR) profiles of urine and plasma samples from pre- and post-operative PCa patients ($n$ = 11) and normal healthy donors (16 urine and 24 plasma) using HTG EdgeSeq miRNA Whole Transcriptome Assay based on next-generation sequencing. Furthermore, tumor-related miRs were detected in formalin-fixed paraffin-embedded tumor tissues obtained from patients with localized PCa. Specific cfmiRs signatures were found in urine samples of localized PCa patients using differential expression analysis. Forty-two cfmiRs that were detected were common to urine, plasma, and tumor samples. These urine cfmiRs may have potential utility in diagnosing early-stage PCa and complementing or improving currently available PCa screening assays. Future studies may validate the findings.

**Keywords:** microRNA; cell-free microRNA; urine; plasma; prostate cancer; diagnosis

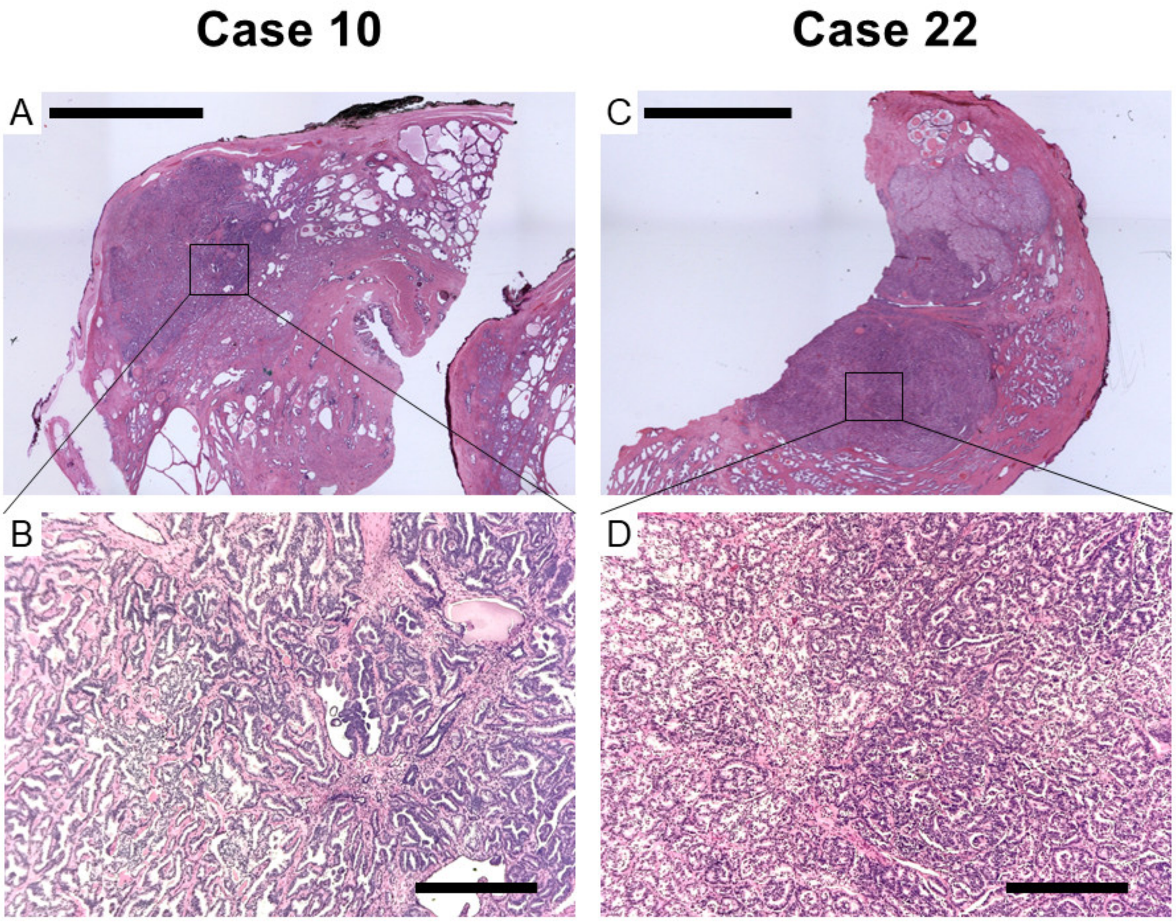


**Supplementary Figure S1.** The hematoxylin and eosin-stained sections of representative pT2 prostate cancer (PCa) tissue samples. Representative images of PCa tumor tissues for two cases; case 10 (**A**,**B**) and case 22 (**C**,**D**) are shown. Case 10 represents grade group 3 and case 22 represents grade group 4. The upper images (**A**,**C**) represent low-power magnification field images (scale bar = 5 mm) and the lower images (**B**,**D**) represent high-power magnification field images (scale bar = 500 µm)**.**
